# Atrial Strain and Strain Rate in a General Population: Do These Measures Improve the Assessment of Elevated NT-proBNP Levels?

**DOI:** 10.1155/2024/1546629

**Published:** 2024-08-26

**Authors:** Assami Rösner, Mikhail Kornev, Hatice Akay Caglayan, Sandro Queiros, Sofia Malyutina, Andrew Ryabikov, Alexander V. Kudryavtsev, Henrik Schirmer

**Affiliations:** ^1^ Department of Cardiology University Hospital of North Norway, Tromsø, Norway; ^2^ Department of Clinical Medicine UiT Arctic University of Norway, Tromsø, Norway; ^3^ Life and Health Sciences Research Institute (ICVS) Escola de Medicina University of Minho, Braga, Portugal; ^4^ ICVS/3B's-PT Government Associate Laboratory, Guimaraes, Braga, Portugal; ^5^ Research Institute of Internal and Preventive Medicine Branch of the Institute of Cytology and Genetics Siberian Branch of the Russian Academy of Sciences, Novosibirsk, Russia; ^6^ Novosibirsk Medical Institute, Novosibirsk, Russia; ^7^ Department of Community Medicine UiT Arctic University of Norway, Tromsø, Norway; ^8^ International Research Competence Centre Northern State Medical University, Arkhangelsk, Russia; ^9^ Department of Cardiology Akershus University Hospital, Lillestrøm, Norway; ^10^ Institute of Clinical Medicine Cardiovascular Research Group Campus Ahus University of Oslo, Oslo, Norway

## Abstract

**Background:**

Noninvasive assessment of elevated filling pressure in the left ventricle (LV) remains an unresolved problem. Of the many echocardiographic parameters used to evaluate diastolic pressure, the left atrial strain and strain rate (LA S/SR) have shown promise in clinical settings. However, only a few previous studies have evaluated LA S/SR in larger populations.

**Methods:**

A total of 2033 participants from Norwegian (Tromsø 7) and Russian (Know Your Heart) population studies, equally distributed by age and sex, underwent echocardiography, including atrial and ventricular S/SR and NT-proBNP measurements. Of these, 1069 were identified as healthy (without hypertension (HT), atrial fibrillation (AF), or structural cardiac disease) and were used to define the age- and sex-adjusted normal ranges of LA S/SR. Furthermore, the total study population was divided into groups according to ejection fraction (EF) ≥50%, EF <50%, and AF. In each group, uni- and multiple regression and receiver operating characteristic curve analyses were performed to test LA and LV functional parameters as potential indicators of NT-proBNP levels above 250 ng/ml.

**Results:**

The mean LA S/SR values in this study were higher than those in previous large studies, whereas the lower references were comparable. In normal hearts, atrial total strain (ATS) and mitral valve E deceleration time (MV DT) were independent factors indicating elevated NT-proBNP levels, whereas in hearts with reduced EFs, the independent indicators were peak atrial contraction strain (PACS) and LV stroke volume. The areas under the curve for these significant indicators to discriminate elevated NT-proBNP levels were 0.639 (95% confidence interval (CI): 0.577–0.701) for normal EF and 0.805 (CI: 0.675–0.935) for reduced EF.

**Conclusion:**

The results confirm good intrastudy reproducibility, with mean values in the upper range of previous meta-analyses. In the future, automated border-detection algorithms may be able to generate highly reproducible normal values. Furthermore, the study showed atrial S/SR as an additional indicator of elevated NT-proBNP levels in the general population, demonstrating the incremental value of both ATS and PACS in addition to conventional and ventricular strain echocardiography. Thus, the LA S/SR may be regarded as an important addition to the multiparametric approach used for evaluating LV filling.

## 1. Introduction

The left atrium (LA) used to be regarded as a simple conduit chamber; however, LA structure and function are directly related to left ventricular (LV) filling pressure. Speckle-tracking echocardiography provides measurements of the deformation of all cardiac chambers over time and has been used for over a decade to measure LA strain and strain rate (S/SR) [1]. LA strain has been shown to reflect different stages of atrial function which can be described as LA peak longitudinal shortening during atrial contraction (PACS), atrial conduit strain (ACS), and total strain (ATS) (or reservoir strain) reflecting the sum of the passive (ACS) and active (PACS) shortening of the atrial wall during the cardiac cycle [2]. Atrial strain has been associated with elevated filling pressures [3–5], possibly indicating diastolic dysfunction [6, 7], heart failure with preserved ejection fraction (HFpEF) [8, 9], the clinical prognosis of different cardiovascular diseases [10, 11], and the prediction of new-onset atrial fibrillation (AF) [3, 4, 12]. Normal LA S/SR values have been established in previous meta-analyses [13] and large population-based studies [14, 15].

NT-proBNP is a well-established marker of LV filling pressure [16]. Noninvasive assessment of diastolic dysfunction, especially in HFpEF, is challenging; therefore, NT-proBNP and echocardiography are the only available screening methods for assessing increased diastolic pressure parameters in heart failure diagnostics.

There are multiple parameters from different echocardiographic modalities indicating elevated filling pressures, where all single measures seem to be suboptimal and only the combination of several parameters is an acceptable indicator. Some studies have shown promising results using LA S/SR for the detection of high filling pressures and NT-proBNP [5, 10].

The 2016 guidelines for the assessment of LV diastolic function have not yet mentioned LA S/SR; however, although they recommend combining different echocardiographic parameters, the sensitivity and specificity were rather low, especially in inconclusive cases. The first artificial intelligence- (AI-) based studies on echocardiographic strain-based parameters [17, 18] supported the inclusion of LA S/SR in a combined approach for the assessment of cardiovascular risk and showed significant improvement by including these parameters.

This study aimed to determine the normal ranges for speckle-tracking imaging-derived atrial S/SR and LA stiffness index (LASI) based on two population studies from Norway and Russia and investigate whether LA S/SR parameters, including LASI, render incremental values in addition to conventional echocardiographic parameters to detect elevated NT-proBNP in the general population. According to the 2016 recommendations [19], the study population was divided into normal, reduced, and AF groups.

This study aimed to establish more robust normal values through a large-scale, population-based evaluation of atrial strain and strain rate parameters, and its novel aspect lies in examining their correlation with NT-proBNP levels.

## 2. Methods

### 2.1. Study Population

The study population comprised participants from the Seventh Tromsø Study (Tromsø7) in Norway and the Know Your Heart (KYH) study in Russia, which are cross-sectional population-based studies. The Tromsø7 study was conducted between March 2015 and October 2016 in the Tromsø municipality, Norway, and the KYH study was conducted from 2015 to 2018 in Arkhangelsk and Novosibirsk, Russia. The Heart-to-Heart study was designed to investigate the causes of increased cardiovascular mortality in the Russian population [20]. Both the H2H and Tromsø7 studies were conducted in parallel, utilizing harmonized questionnaires, health examinations, biological sample collection, and echocardiography protocols.

**Figure 1:**
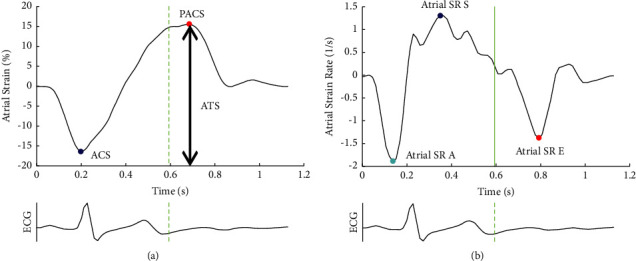
Example of global atrial strain and SR curves for the extraction of peak values. The cycle starts at the end of the P-wave. PACS, peak atrial contraction strain; ACS, atrial conduit strain; ATS, atrial total strain; SR, strain rate; S, during systole; E, during the early filling phase; A, during atrial contraction. (a): EF ≥ 50%. EF, ejection fraction; ATS, atrial total strain; MV DT, mitral valve deceleration time. (b): EF < 50%. PACS, peak atrial contraction strain; LVSV, left ventricular stroke volume; ESV, end-systolic volume.

In the Tromsø7 study, the inclusion age range was 40 years and older without an upper limit, whereas the KYH study included participants aged 35–69 years. Speckle-tracking analysis was performed to compare both populations [21], and echocardiograms were selected from approximately equal-sized age and sex groups from both populations (40–49, 50–59, and 60–69 years). The final sample for the present study comprised about equal sized groups from Norway and Russia, with 46% from Tromsø and 27% each from Arkhangelsk and Novosibirsk.  Figure 1 shows a flowchart of the number of participants included and excluded from this study and their division into study groups.

### 2.2. Definition of Normalcy

To define normal ranges in healthy individuals, atrial S/SR-based parameters were investigated in selected individuals. For this purpose, participants were excluded if one or more of the following criteria were met: moderate-to-severe valvular heart disease and history or objective indicators of previous coronary artery disease (classes 1.1–1.2.7 of the Minnesota Code), cardiomyopathies or other states of reduced ventricular function, electrocardiogram (ECG) with a QRS complex more than 130 ms, ejection fraction (EF) less than 50%, hypertension (HT), antihypertensive medication, history of AF, AF on ECG, or elevated NT-proBNP.

HT was defined as a systolic blood pressure (BP) ≥140 mmHg and/or diastolic BP ≥ 90 mmHg during the visit, regular intake of anti-hypertensive drugs, or a reported history of HT. All study participants were asked about their current medications, and the data was coded using the Anatomical Therapeutic Chemical (ATC) classification system. Any medication within the ATC classes C02, C03, C07, C08, or C09 was regarded as an antihypertensive. Elevated NT-proBNP values were homogenized between the Norwegian and Russian study groups [22], and a cutoff value of 250 ng/ml was chosen according to the average upper normal range (99 percentile) based on a study in the Tromsø7 population [23].

### 2.3. Data Collection and Echocardiography in Tromsø7 and KYH

Transthoracic echocardiography was performed in the left lateral decubitus position using commercially available GE Healthcare systems, in Tromsø7 using a high-end Vivid E9 ultrasound system with a single crystal matrix sector probe of 1.5–4.6 MHz, while the KYH was performed on a Vivid Q machine with a 1.5–3.6 MHz sector matrix transducer. Conventional acquisitions, including two-dimensional (2D) grayscale images and M-mode pulsed, continuous, and color Doppler data, were performed in the parasternal and apical views. 2D-images were obtained at a frame rate of at least 50 Hz. In both countries, experienced readers utilized similar EchoPAC workstations (v.113; GE-Vingmed AS, Horten, Norway). The offline analysis in Norway utilized one ECHO reader (MS), while in Russia it was conducted by three ECHO specialists (S.M., An.R., and V.G.). Intra- and interobserver variability for conventional echocardiographic measures was regularly assessed within both the Tromsø7 and KYH reading laboratories and compared between laboratories. Conventional echocardiography included LV systolic and diastolic volumes (LV ESV and LV EDV, respectively) and LV outflow tract (LVOT) Doppler-derived stroke volume (SV). EF as well as LA volumes were calculated using the 2D Simpson biplane method. The Doppler-derived measurements included mitral valve (MV) E and A, E/A ratio, and deceleration time (MV DT). The M-mode was used to calculate the myocardial mass and LVOT diameter for the SV calculation. SV, myocardial mass, and LA volume were indexed using body surface area (BSA). Conventional readings were performed by two reading laboratories in Norway and Russia, and LA volume was estimated using a single reader (M.K.). In accordance with the 2016 recommendations for assessment of diastolic function [19], we defined “indeterminate” when 2 out of the 4 the following criteria were met: E/e´ > 14, septal e´ velocity <7 cm/s or lateral e´ velocity <10 cm/s, TR velocity >2.8 m/s and LA volume index (LAVI) > 34 ml/m^2^, and definite diastolic dysfunction, when >2 out of 4 of these criteria were met. Elevated filling pressures were identified when E/A ≥2 classified as diastolic dysfunction grade III, when E/A ≤ 0.8 and E ≥ 50 cm/s, or when E/A was >0.8 but <2, the presence of elevated filling pressures or grade II diastolic dysfunction was determined if two or three of the following criteria were met: average *E*/e' ≥ 14; TR velocity ≥2.8 m/s and LA vol. index ≥34 ml/m^2^.

### 2.4. S/SR Analysis

For LV S/SR, a single reader (M.K.) analyzed the 2D apical four- and two-chamber and apical long-axis (APLAX) views and dedicated two- and four-chamber views for atrial volume and atrial S/SR with speckle tracking using the Q-analysis function of EchoPAC (v.203, GE-Vingmed AS, Horten, Norway). The LV S/SR from APLAX views was analyzed in 176 Tromsø7 participants and the global S/SR was derived from the four- and two-chamber views only.

**Figure 2:**
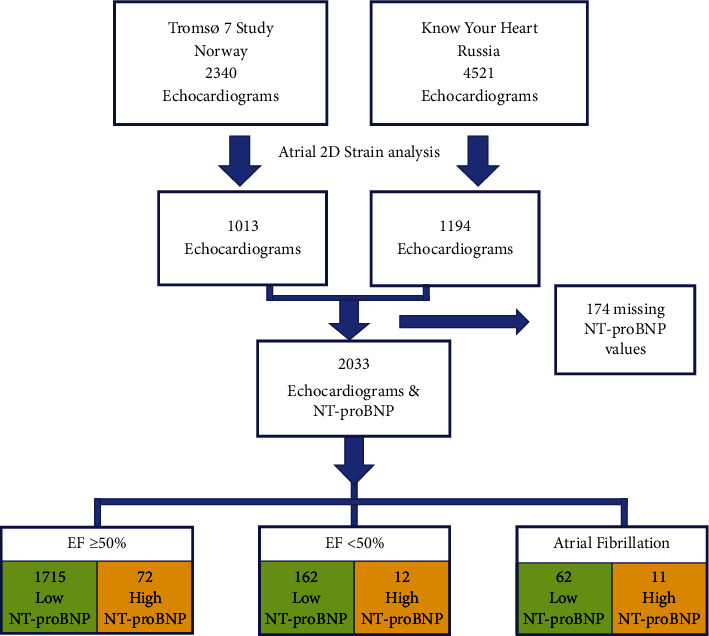
Flowchart of participant inclusion, selection, and distribution into different groups.

Cycles for LV strain analysis were set to start at peak R, whereas the cycle for atrial contraction was defined as the end of the P-wave. The end of P-wave was chosen to get as close as possible to the time-point of onset of active atrial contraction. In AF, peak R was defined as the onset of the cardiac cycle. Aortic valve closure was defined by using a transaortic CW Doppler signal. For atrial and ventricular measurements, the region of interest (ROI) was manually traced at the subendocardial border, with consecutive adjustments in the ROI width. Automated tracking was visually controlled and suboptimal tracking results were repeated a maximum of three times. For LV and LA strains, segmental values were extracted from longitudinal mid-myocardial strain curves averaged over the number of segments. The LV strain was measured at the time of ES. As shown in Figure 2, the LA S/SR was measured from the global strain curves derived from the strain and SR curves of all six segments of the four-chamber view. As the Q-analysis function did not provide all peak atrial S/SR measurements for the defined time periods, the results were automatically extracted from the strain curves using customized software. Although atrial strains reflect longitudinal shortening and should be expressed as negative values, there appears to be a general consensus on reporting atrial longitudinal strain values as positive numbers. LA strain was measured in three defined time periods: peak atrial contraction strain (PACS) between the onset of atrial contraction and peak negative strain; ATS, defined as the difference between the negative and positive peaks; and ACS, defined as the difference between the positive peak and the onset of atrial contraction. In the LA and LV, diastolic SR E was measured at peak SR after AVC and before the onset of the atrial contraction, SR during atrial contraction (A) was the SR peak after the onset of the atrial contraction, and systolic SR during systole (S) was the peak in the opposite direction between the start of the cycle and AVC. Tissue Doppler velocities from the septum and lateral wall were derived from basal segmental speckle tracking analyses, which were chosen to overcome the differences between the two study populations in terms of tissue Doppler acquisitions with different ultrasound systems and machine settings. Mitral E/e´ was calculated from the MV E and the average basal and lateral four-chamber e´ was calculated from the basal speckle tracking-derived velocities. LASI was calculated as the ratio of the E/e´ and ATS, where e´ was derived from the basal velocities of the septum and lateral wall in the four-chamber view.

### 2.5. Statistical Analyses

Statistical analyses were performed using IBM SPSS version 28.0. (IBM Corp.: Armonk, NY, US). The two groups were compared using either the *t*-test or *χ*^2^ test for continuous and categorical variables, respectively. Unless otherwise stated, continuous variables are presented as means ± standard deviations (SD). Variables with skewed distributions are presented as medians with quartiles (Q1/Q3). Categorical characteristics are presented as absolute numbers and proportions (%). For comparison between the three groups with EF more than or equal to 50% (EF ≥ 50%), EF less than 50% (EF < 50%), and AF, group differences in continuous variables were tested using one-way analysis of variance (ANOVA) with Bonferroni post hoc tests.

For the independent and dependent correlations of echocardiographic indices with the presence of elevated NT-proBNP levels, univariate and multivariate logistic regression analyses were performed. Variables with a *p* value of less than or equal to 0.20 in the univariate analysis were selected and tested by forward and backward multivariate logistic regression analysis. For final inclusion in the multiple regression model, a *p* value of less than or equal to 0.05 was considered statistically significant. Independent predictors of the multivariate analysis were combined as weighted predictors and a receiver operating characteristic (ROC) curve analysis was performed.

### 2.6. Intra- and Interobserver Variability

For intra-and interobserver variability in atrial S/SR measurements, the same observer repeatedly analyzed 45 randomly selected echocardiographic records. The same data was reanalyzed by a second experienced observer. Intra- and interobserver values were calculated as intraclass correlations and 95% confidence intervals (CIs).

## 3. Results

The characteristics of the participants are presented in Table 1. Participants were divided into two groups based on their NT-proBNP levels being above or below 250 pg/ml with high NT-proBNP levels being observed in 4.6% of patients in the selected cohort. High NT-proBNP levels were more prevalent in men and in a high proportion of the Russian population. Participants with high NT-proBNP levels were significantly older and had larger weights, body mass indexes, higher glycosylated hemoglobin levels, creatinine levels, and high-sensitive C-reactive protein levels. Furthermore, participants with higher NT-proBNP levels displayed a higher percentage of cardiac pathology (by Hx, echocardiogram, or ECG), whereas the majority (85.3%) of participants in this group had no cardiac diagnosis; however, 71% were taking antihypertensive drugs, indicating a high prevalence of HT. Low-density lipoprotein (LDL) and total cholesterol levels were lower, probably because of the higher prescription rate of lipid-lowering drugs in this group.

Table 2 presents age- and sex-related normal ranges for atrial strain and SR values in a selection of 1069 healthy individuals (619 females and 450 males). ACS, ATS, atrial SR E was significantly dependent on age and sex, being higher with age and reduced in males. LASI increased with age and male sex, while PACS SR S and SR A showed no significant age- and sex-related changes.

Systolic and diastolic conventional and strain derived atrial and ventricular parameters are listed in Table 3 grouped by EF ≥ 50%, EF < 50% and AF. In each group, we aimed to identify the indicators of high and low NT-proBNP levels.

In all groups, NT-proBNP was highly correlated with lower atrial S/SR, higher LASI, some indicators of impaired relaxation such as lower basal E velocity and LV PL, SR, and E, and indicators of higher filling pressures such as higher E/e´. The E/A ratio was higher only in the AF group, and systolic parameters were significantly lower only in the EF < 50% group. In the EF ≥ 50% group, EDV and ESV were slightly, but insignificantly, reduced, and the LV mass was higher, indicating the presence of LV hypertrophy in subclinical heart failure with normal EF.

The mean values and differences between the three groups are shown in Table S1 of the supplementary material. Compared with the EF ≥ 50% group, the EF < 50% and AF groups displayed lower atrial and ventricular diastolic S/SR and velocities. LASI increased with reduced EF and AF, whereas the Doppler-derived diastolic functional parameters in AF were not significantly different. However, parameters with one-sided changes during diastolic dysfunction, like tricuspid regurgitation peak gradient, E/e´, and LA volume, indicated significant diastolic dysfunction of the low EF ventricles with AF.

Following the 2016 ESC recommendations for evaluation of diastolic function, we identified that within the normal group, 22 individuals (2.3%) showed indeterminate characteristics, and none (0.0%) had clear characteristics of diastolic dysfunction. In contrast, among 661 individuals with treated or untreated hypertension, 81 individuals (12%) showed an indeterminate, and 9 individuals (1.4%) exhibited clear characteristics of diastolic dysfunction while 114 (17%) had elevated NT-proBNP levels. However, only 11 of these 114 individuals (9.6%) were categorized by echocardiography as having indeterminate characteristics for diastolic dysfunction, and just 2 individuals (1.8%) were identified as having a high probability of elevated filling pressures according to the 2016 recommendations. In total, only 14 individuals met the criteria for grade II diastolic dysfunction and elevated filling pressures and 2 individuals met the criteria for III. Thus, no significant correlation between the 2016 criteria for elevated filling pressures and NT-proBNP levels could be demonstrated.

**Figure 3:**
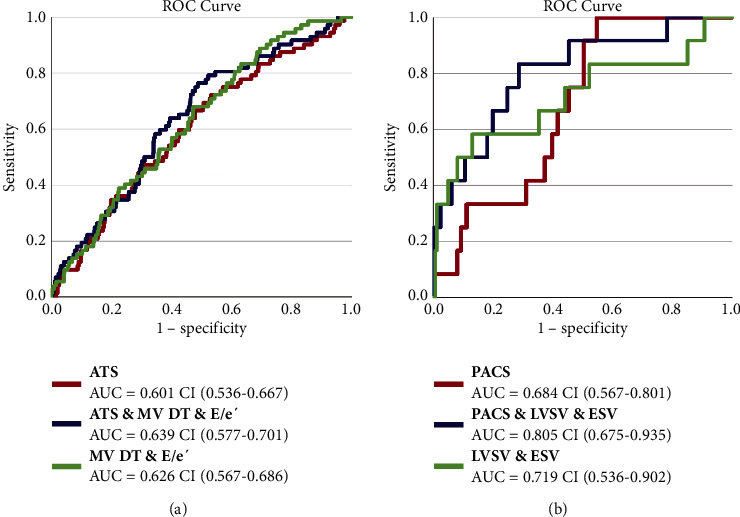
Receiver operating characteristic curves for indicators of elevated NT-proBNP.

Tables S2, S3, and S4 display univariate and multiple binary logistic regression analyses for systolic and diastolic echocardiographic parameters in relation to normal and high NT-proBNP levels for the EF ≥ 50% (Table S2), EF < 50% (Table S3), and AF (Table S4) groups. In participants with EFs more than or equal to 50% (Table S2), univariate regression showed a large number of diastolic and systolic parameters as indicators for the presence of high NT-proBNP levels. Multiple regression analysis revealed that ATS (HR = 0.978, CI = 0.958–0.998, *p*=0.031) and MV-DT (HR = 1.007, CI = 1.003–1.012, *p*=0.002) were the strongest independent indicators of a normal EF. In the EF < 50% group (Table S3), the results of the univariate analysis were similar, whereas PACS (HR = 1.44, CI = 1.10–1.87, *p*=0.007), LV SV (HR = 0.91, CI = 0.85–0.98, *p*=0.010), and LVESV (HR = 1.06, CI = 1.01–1.11, *p*=0.011) were independent indicators of the presence of high NT-proBNP by multiple binary regression. In the smallest group with AF (Table S4), only univariate regression was performed because the number of positive cases was too low for a reliable result in the multiple regression analysis. Similar to hearts without AF, all atrial S/SR parameters and many systolic and diastolic parameters indicated the presence of high NT-proBNP levels.  Figure 3 shows the ROC curves for both groups with low and normal EF for the combination of parameters of the multiple regression analyses discriminating the presence of high NT-proBNP levels. For normal EF, the combination of parameters did not significantly increase the area under the curve (AUC), whereas adding PACS to MV DT and ESV significantly increased the AUC from 0.719 to 0.805. The intra- and interobserver variability by intraclass correlation shown in Table S5 shows acceptable to good reproducibility for all atrial S/SR parameters.

The supplemental material includes figures that illustrate the correlation between the various atrial S/SR values and NT-proBNP levels, specifically Figure S1 for individuals with HT and Figure S2 for those with a history of AF. It was observed that low atrial S/SR values correlate with both high and low NT-proBNP levels, whereas high atrial S/SR values were not associated with high NT-proBNP levels. Furthermore, the correlation between AF and elevated NT-proBNP was more pronounced than in the HT group.

## 4. Discussion

This study showed normal ranges of atrial S/SR in two large population-based studies. Furthermore, it demonstrated that reduced PACS and ATS were related to high NT-proBNP levels and these parameters had incremental value in the detection of elevated NT-proBNP in the general population.

### 4.1. Normal Ranges for Atrial Strain and SR

Several previous studies have reported normal values and ranges for atrial S/SR; however, only a few publications have referred to larger population-based studies with more than 1000 healthy subjects [14, 15]. Apart from the inclusion of participants with diabetes in the present study, the exclusion criteria were similar in all the three studies. In accordance with these two large previous studies, our data confirmed higher ACS, ATS, and SRE in the younger population and in females. Nielsen et al. reported an increase in PACS with age, whereas our results showed the same but not a significant tendency. The absence of age dependence in SR S and SR A reflects previous findings [14].

Compared with the population-based studies of Nielsen et al. [15] and Liao et al. [14] (1641 and 2812 healthy participants, respectively), the mean atrial strain values in the present study were generally higher. They were also generally higher than those in the meta-analysis by Pathan et al.:23% vs. 21%, 27% vs. 23%, and 47.5% vs. 39% for PACS, ACS, and ATS, respectively. The meta-analysis showed high variations between the underlying studies, with a range of ATS between 28% and 60%, illustrating the challenges of defining normalcy in thin-walled atria. The high range may be due to difficulties in defining the atrial wall, which must be defined at the longest distance from the probe, leading to a low lateral resolution of the atrial walls. Small changes in the ROI position and the definition of atrial wall thickness (as necessary in EchoPac) can significantly affect the outcomes of atrial S/SR measurements. Large differences between studies and the significance of relatively small differences within each study indicate systematic errors between different laboratories.

For clinicians, the most important values for defining normalcy are the lower limits. Interestingly, compared with the study by Nielsen et al. [15], our study showed that PACS and ATS were similar, whereas the lower limits for ACS were lower, with higher variations than those for PACS. Similarly, the lower limits may be explained by the higher values in the present study, with higher data variability. Comparing SR S, SR E, and SR A between the present study and the one by Liao et al. [14], we found similar mean values for SR S and SR A (2.5 vs. 2.6; 2.3 vs. 3.0; and 3.0 vs. 2.9/s, respectively), while SR E was lower (2.3 vs. 3.0, respectively). For the SR, the SD was larger (0.9/s) than that of the study by Liao et al. (0.5/s). The higher SD may reflect a higher variability between the Norwegian and Russian populations, apart from the lower population size and lower imaging or reading quality. Higher values of atrial strain may be due to a more central position of the ROI which might also explain the higher variability of the measurements.

### 4.2. Normal EF

Several studies have confirmed the usefulness of NT-proBNP as a marker of elevated filling pressure, indicating the presence of subclinical heart failure in asymptomatic patients [16, 24, 25]. Other studies have shown a relationship between adverse outcomes and HFpEF and heart failure with reduced EF (HFrEF) [26, 27]. The cut-off value for a normal NT-proBNP in the present study has been chosen by averaging the 99 percentile of recently published age- and sex-adjusted normal-ranges of the Tromsø Study [23].

A recent population-based study of 620 individuals with normal EFs investigated LA S/SR and LASI in relation to elevated NT-proBNP levels [28] and showed weak but significant correlations between S/SR parameters and elevated NT-proBNP levels. In accordance with the study by Liu et al., all three groups with normal EF, low EF, and AF showed significantly lower atrial S/SR at elevated NT-proBNP levels, confirming the previous numbers with three times as much participants.

Based on the 2016 recommendations for hearts with preserved EF, E/e,' septal e,' lateral e´, TR velocity, and LA volume should be used to assess elevated filling pressures. In the normal EF group of the present study, *E*/e' and MV DT, in addition to ATS, were independently associated with elevated NT-proBNP levels. However, these differing results cannot be regarded as a contradiction since larger studies on diastolic function have so far not been able to show sufficient test accuracies for single parameters, with the highest AUCs of around 0.7. Given the small effect sizes of each possible indicator, multiple regression analyses in different studies always favor different parameters. Liu et al. showed that LASI is a good marker of high NT-proBNP levels, which was confirmed in the present study. However, LASI was not a better indicator than the other atrial S/SR parameters.

### 4.3. Reduced EF

Participants with reduced EF and normal NT-proBNP are a diverse group, where either systolic or diastolic heart function is within the low-normal range, echocardiographic pathology precedes an increase in NT-proBNP, and participants may present at the stage of compensated heart failure. Thus, this study population cannot be compared with clinical studies, in which extreme values render higher test accuracies. However, because the 2016 recommendations for the assessment of diastolic dysfunction suggest a different approach for the assessment of diastolic filling pressures in hearts with reduced EFs [19], we chose to investigate the subpopulation with reduced EF separately.

Although the group with reduced EF comprised only 173 participants and 12 pathological cases, atrial S/SR revealed similar results to the normal EF group. As in the high EF group, independent conventional indicators for high NT-proBNP such as LV SV and LV ESV were different from the recommended parameters (E/A ratio, MV E, E/e´, TR velocity, and LAVI) from the 2016 guidelines [19]. According to the multiple regression, PACS was an “independent” indicator for elevated NT-proBNP, supporting previous reports, that atrial S/SR are valuable indicators for high filling pressures and diastolic dysfunction [6–8]. Accordingly, ROC curve analysis revealed a significant effect of adding these parameters to the combination of two conventional parameters, LVSV and LVESV. Interestingly, PACS was the only independent diastolic functional indicator of high NT-proBNP levels, indicating the usefulness of adding atrial S/SR parameters to the assessment of diastolic dysfunction.

### 4.4. AF

Previous studies demonstrated that LA S/SR is a useful predictor of the incidence of recurrent AF [3, 4, 29], LA reverse remodeling after radiofrequency catheter ablation [30], and future embolic events [31]. However, this population-based study included only a small group of participants with AF, and unexpectedly, the number of participants with elevated NT-proBNP levels was low. In addition, this group was highly inhomogeneous, comprising participants with sinus rhythm, a history of AF, and presenting with AF during echocardiographic examination. The small group size and differences between registrations with sinus rhythm or AF were the most likely causes of the insignificant outcomes of the multiple regression analysis. However, a significant indirect comparison of groups and univariate regression analysis revealed the highest number of possible predictors of increased filling pressures, including atrial S/SR parameters, especially PACS and atrial SR, which is in accordance with previous studies confirming the importance of LA S/SR in assessing cardiac function and outcomes.

### 4.5. Clinical Application

Invasive catheter-based studies have shown that the atrial S/SR is a valuable indicator of high filling pressures [5, 32]. However, this was a population study based on healthy or asymptomatic participants to ascertain whether echocardiographic screening for elevated filling pressures or heart failure could be improved by adding LA S/SR parameters as previous studies have shown that high clinical values of atrial S/SR and LASI may predict outcomes in patients with previous AF [3, 4, 30, 31].

Assessing elevated filling pressures without an invasive approach remains challenging. Although the European Association of Cardiovascular Imaging (EACVI) recommendations suggest a limited number of parameters, modern echocardiography provides over 40 Doppler, tissue Doppler, and speckle tracking-based parameters that may be used to evaluate LV filling pressures. Pandey et al. introduced a deep-learning AI-based approach that included LA S/SR, 13 echocardiographic measures, and clinical parameters [17]. All current approaches indicate that a single key parameter cannot accurately assess diastolic dysfunction, especially in asymptomatic patients. Furthermore, AI-based assessment and a clinician visually assessing the echocardiograms will still both be needed to assess many accessible parameters for optimal reading results, especially in large areas of inconclusive or contradictory measurements as the present study showed that LA strain adds incremental information; therefore, these parameters should be integrated into the assessment of LV filling pressure.

### 4.6. Limitations

This study has several limitations. According to previous meta-analyses and reviews [13, 33], LA strain measurements and variability differed significantly between the study groups. Furthermore, this was a single-reader study with low intrareader variability. Multiple readers should be involved to test for a screening situation. Furthermore, using strain-values from two different types of ultrasound scanners, might influence the strain-results. Values of normalcy might need to adjusted for different vendors and software.

Another problem is that normalcy included individuals with high-risk factors for cardiovascular disease like high cholesterol, obesity, and diabetes. The latter two are known to influence the diastolic properties of the left heart. However, excluding all participants with cardiovascular risk factors, manifest heart disease, and hypertension leaves only 18% of the study population as “normal individuals,” which does not represent average measurements in a generally healthy population. The cohort of “healthy individuals” still included only 39% of study-participants, but seemed to be more appropriate to represent “normalcy” than the “super-normal” 18%. However, we could show in an earlier study that there were only minor differences in the study cohort with- or without cardiovascular risk-factors [21].

General population-based studies have limitations in reproducing the test accuracies observed in selected patients with symptoms or a high-risk profile. However, this study was able to answer the question of the incremental value of LA S/SR compared with conventional echocardiographic parameters. Averina et al. [23] demonstrated that NT-proBNP has low sensitivity and specificity for detecting cardiac disease in the general population, reflecting the challenge of evaluating the test accuracy within only low pathological deviations. Furthermore, the chosen NT-proBNP cutoff value plays a crucial role in test accuracy. Cardiac disease can be present at high or low filling pressures; therefore, the question remains whether NT-proBNP is a sufficient indicator of filling pressures in the general population.

## 5. Conclusion

Creating reliable reference values for LA S/SR is challenging, and the present results confirm good intrastudy reproducibility, with mean values in the upper range of previous meta-analyses. In the near future, automated border detection algorithms may be required to generate highly reproducible normal values. Furthermore, our study showed atrial S/SR as an additional indicator of elevated NT-proBNP levels in the general population, demonstrating the incremental value of both ATS and PACS in addition to conventional and ventricular strain echocardiography. Thus, the LA S/SR may be regarded as an important addition to the multiparametric approach used for evaluating LV filling.

## Figures and Tables

**Table 1:** Group characteristics for participants grouped by low and high NT-proBNP.

	Low NT-proBNP	High NT-proBNP	*p* value
	Mean ± SD, median (QR) or *n* (%)	Mean ± SD, median (QR) or *n* (%)	
Group *n*	1939	95	
Women	1002 (52)	42 (44)	0.155
Men	937 (48)	53 (56)	
Norwegian	849 (44)	25 (26)	<0.001
Russian	1090 (56)	70 (74)	
Age (years)	55.2 ± 8.5	58.1 ± 8.3	0.001
Height (cm)	170 ± 10.4	170 ± 9.5	0.597
Weight (kg)	79.6 ± 16.5	83.2 ± 21	0.039
BMI (kg/m^2^)	27.7 ± 5.2	26.8 ± 5.2	0.038
Systolic BP (mmHg)	131 ± 20	135 ± 21	0.078
Diastolic BP (mmHg)	79.9 ± 11.8	82.3 ± 13.3	0.061
LDL cholesterol (mmol/l)	3.7 ± 0.9	3.3 ± 1.0	<0.001
Cholesterol (mmol/l)	5.6 ± 1.1	5.1 ± 1.2	<0.001
Antihypertensive drugs (*n*)	643 (29)	41 (71)	<0.001
HbA1C (%)	5.6 ± 0.7	5.9 ± 0.9	0.001
Diabetes (*n*)	103 (5.3)	9 (9.5)	0.083
Smoking daily (*n*)^∗∗^	1061 (55)	54 (57)	0.685
Creatinine (mmol/l)	80.1 ± 16.1	103.2 ± 88.3	<0.001
HS CRP (mg/l)^‡^	1.26 (0.6/2.7)	2.8 (1.1/5.3)	<0.001
NT-proBNP (pg/ml)^‡^	52.6 (30.9/85.8)	373 (300/699)	<0.001
Cardiac pathology (*n*)	202 (10.4)	14 (14.7)	0.04

BMI: body mass index; BP: blood pressure; LDL: low-density lipoproteins; HbA1C: glycosylated hemoglobin. HS CRP: high sensitive C-reactive protein; cardiac pathology by Hx, echocardiogram or ECG. ^‡^NT-proBNP and HS CRP: median with quartiles. ^∗∗^Refers to present and previous smoking.

**Table 2:** Mean and normal ranges for males and females in different age-groups.

Age group	40–49	50–59	60–69

PACS (%)			
Female	20.1 (9.0 to 31.2)	20.5 (9.4 to 31.6)	21.0 (9.7 to 32.3)
Male	20.4 (8.9 to 31.9)	20.4 (9.3 to 31.3)	21.6 (8.6 to 34.7)
ACS (%)			
Female	34.9 (6.4 to 63.3)	28.1 (5.2 to 50.9)	24.4 (2.2 to 46.5)^†^
Male	27.4 (2.9 to 51.9)^‡^	23.8 (4.0 to 43.6)^‡^	22.7 (3.6 to 41.9)
ATS (%)			
Female	54.0 (22.2 to 87.7)	48.6 (21.1 to 76.1)	45.4 (17.6 to 73.2)
Male	47.8 (17.8 to 77.9)^‡^	44.1 (18.6 to 69.7)^‡^	44.3 (19.4 to 69.3)
Atrial SR S (1/s)			
Female	2.7 (0.8 to 4.5)	2.4 (0.8 to 4.1)	2.4 (0.6 to 4.3)
Male	2.6 (0.5 to 4.7)	2.3 (0.8 to 4.1)	2.5 (0.7 to 4.4)
Atrial SR E (1/s)			
Female	−3.0 (−0.8 to −5.3)	−2.4 (−0.6 to −4.2)	−1.9 (−0.5 to −3.2)^†^
Male	−2.5 (−0.4 to −4.7)^‡^	−2.2 (−0.6 to −3.9)^‡^	−2.0 (−0.4 to −3.6)
Atrial SR A (1/s)			
Female	−2.9 (−1.2 to −4.6)	−2.9 (-1.1 to −4.6)	−2.9 (−1.0 to −4.9)
Male	−3.0 (−0.9 to −5.1)	−3.0 (−0.9 to −5.0)	−3.1 (−1.0 to −5.2)
LASI (1/%)			
Female	0.17 (0.06 to 0.35)	0.19 (0.09 to 0.41)	0.24 (0.10 to 0.58)^†^
Male	0.18 (0.07 to 0.37)^‡^	0.19 (0.08 to 0.42)	0.21 (0.09 to 0.54)^‡^

Measurements of healthy individuals with EF ≥ 50%, without cardiac disease, atrial fibrillation, controlled, or uncontrolled hypertension. PACS: peak atrial contraction strain; ACS: atrial conduit strain: ATS: atrial total strain; SR: strain rate; S: during systole; *E*: during diastole; A: during atrial contraction. ^‡^*p* < 0.05 for difference between males and females. ANOVA with Bonferroni post hoc analysis. ^†^*p* < 0.05 for difference towards group 40–49 years. No significant differences were seen between the age group 50–59 years and other age groups.

**Table 3:** Difference between systolic and diastolic ventricular and atrial function comparing normal with elevated NT-proBNP groups for systolic and diastolic functional parameters.

	EF ≥ 50%		EF <50%		Atrial fibrillation	
	Group A		Group B		Group C	
*n*	1716	72	162	12	61	11
	Low NT-proBNP	High NT-proBNP	Low NT-proBNP	High NT-proBNP	Low NT-proBNP	High NT-proBNP
	Mean ± SD	Mean ± SD	Mean ± SD	Mean ± SD	Mean ± SD	Mean ± SD

PACS (%)	20.6 ± 6.0	19.8 ± 5.9	18.3 ± 7.1	13.8 ± 5.2^∗^	18.5 ± 7.2	10.5 ± 8.4^∗^
ACS (%)	26.1 ± 12.7	22.0 ± 10.1^∗^	19.4 ± 10.4	15.2 ± 7.0	23.3 ± 14.1	16.7 ± 12.0
ATS (%)	46.7 ± 15.2	42.0 ± 13.3^∗^	37.6 ± 13.9	29.0 ± 10.9^∗^	41.7 ± 18.1	27.1 ± 17.4
Atrial SR S (1/s)	2.45 ± 0.97	2.26 ± 0.92	2.09 ± 0.89	1.41 ± 0.62^∗^	2.12 ± 0.90	1.35 ± 0.93
Atrial SR E (1/s)	−2.25 ± 1.03	−1.98 ± 0.92^∗^	−1.67 ± 0.73	−1.27 ± 0.45	−1.90 ± 0.99	−1.46 ± 0.85
Atrial SR A (1/s)	−2.90 ± 0.98	−2.76 ± 1.17	−2.66 ± 1.09	−1.90 ± 0.81	−2.58 ± 1.12	−1.38 ± 0.95^∗^
LASI (1/%)	0.23 ± 0.16	0.28 ± 0.17^∗^	0.34 ± 0.27	0.45 ± 0.25	0.41 ± 1.04	1.06 ± 1.73
MV *E *(cm/s)	67.2 ± 16.2	64.8 ± 16.5	60.8 ± 15.7	54.0 ± 14.6	67.4 ± 21.3	83.2 ± 51.4
MV A (cm/s)	64.1 ± 15.4	64.6 ± 17.3	68.2 ± 16.1	71.2 ± 12.4	66 ± 17	62 ± 26
MV E/A (1/1)	1.10 ± 0.36	1.05 ± 0.33	0.93 ± 0.33	0.76 ± 0.15	1.05 ± 0.35	1.60 ± 1.05^∗^
MV DT (ms)	190 ± 47	211 ± 50^∗^	198 ± 48	182 ± 43	182 ± 47	190 ± 62
LA volume index (ml/m^2^)	22.7 ± 8.4	23.6 ± 10.9	21.5 ± 8.0	23.2 ± 11.9	23 ± 11	37 ± 24^∗^
E/e´ ()	9.67 ± 3.22	10.5 ± 3.98^∗^	11.0 ± 4.3	12.8 ± 4.7	10.8 ± 5.8	12.7 ± 11.0
TAPSE (cm)	2.39 ± 0.40	2.33 ± 0.42	2.25 ± 0.41	1.91 ± 0.59^∗^	2.42 ± 0.53	2.02 ± 0.50^∗^
TR PG (mmHg)^‡^	16.2 (6/20)	16.5 (12/20)	14.3 (3.8/17)	14.9 (1.7/19)	17.4 (3.1/17)	21.7 (13/24)
Velocity basal septal E (cm/s)	−6.5 ± 2.1	−5.9 ± 1.8^∗^	−5.2 ± 1.8	−4.5 ± 1.6	−6.0 ± 2.1	−6.3 ± 2.5
Velocity basal lateral E (cm/s)	8.4 ± 2.7	7.7 ± 2.6^∗^	−6.5 ± 2.6	−5.2 ± 2.6	−7.6 ± 2.9	−7.9 ± 2.9
Velocity basal septal A (cm/s)	−7.5 ± 1.7	−7.6 ± 1.7	−7.2 ± 1.8	−6.7 ± 2.3	−6.8 ± 2.5	−5.0 ± 2.8^∗^
Velocity basal lateral A (cm/s)	−6.9 ± 2.5	−7.4 ± 2.4	−6.3 ± 3.0	−6.0 ± 3.0	−6.5 ± 2.8	−4.7 ± 3.1^∗^
LV Stoke volume (ml)	85.7 ± 22.2	82.2 ± 20.1	84.8 ± 22.0	71.6 ± 14.6^∗^	91.6 ± 24.1	88.8 ± 12.0
LV EF (%)	56.8 ± 5.5	56.3 ± 4.3	43.3 ± 5.2	39.9 ± 9.9^∗^	55 ± 7	49 ± 9^∗^
LV EDV (ml)	129 ± 32	122 ± 39.7	140 ± 40	151 ± 41	140 ± 35	152 ± 64
LV ESV (ml)	43 ± 16	40 ± 23	56 ± 27	79 ± 46^∗^	48 ± 18	64 ± 61
LV mass index (g/m^2^)	119 ± 36	123 ± 39	130 ± 40	120 ± 256	128 ± 40	134 ± 25
LV ES long strain (%)	−20.3 ± 2.7	−19.7 ± 2.7	−17.7 ± 3.1	−15.0 ± 5.9^∗^	−19.9 ± 4.1	−18.4 ± 6.2
LV PL SR S (1/s)	−1.20 ± 0.20	−1.19 ± 0.20	−1.12 ± 0.21	−0.95 ± 0.38^∗^	−1.17 ± 0.23	−1.21 ± 0.34
LV PL SR E (1/s)	1.56 ± 0.34	1.43 ± 0.35^∗^	1.29 ± 0.33	1.09 ± 0.34^∗^	1.55 ± 0.44	1.54 ± 0.60
LV PL SR A (1/s)	1.11 ± 0.27	1.16 ± 0.26	1.02 ± 0.36	1.13 ± 0.32	1.09 ± 0.29	0.86 ± 0.27

PACS: peak atrial contraction strain; ACS: atrial conduit strain; ATS: atrial total strain; SR: strain rate; S: during systole; *E*: during early filling phase; A: during atrial contraction; LASI: left atrial stiffness index (E/E´/LA strain); MV: mitral valve; DT: deceleration time; LA: left atrium; TAPSE: tricuspid annular plane systolic excursion; TR PG: tricuspid regurgitation peak gradient; LV: left ventricle; EF: ejection fraction; EDV: end diastolic volume; ESV: end systolic volume; ES: end systolic; PL: peak longitudinal. ^∗^Pairwise comparisons with *p* < 0.05 for difference between groups with normal and high NT-proBNP; ^‡^median (IQR).

## Data Availability

The data supporting the findings of this study are available from the Know Your Heart and the Tromsø Study; however, restrictions apply to its availability as it was used under license for the current study, and hence, it is not publicly available.

## Abbreviations

A: During atrial contraction
ACS: Atrial conduit strain
AF: Atrial fibrillation
ATS: Atrial total strain
DT: Deceleration time
E: During early filling phase
EDV: End diastolic volume
EF: Ejection fraction
ES: End systolic
ESV: End systolic volume
HT: Hypertension
LA: Left atrium
LASI: Left atrial stiffness index
LV: Left ventricle
MV: Mitral valve
PACS: Peak atrial contraction strain
PL: Peak longitudinal
S: During systole
S/SR: Strain and strain rate
SR: Strain rate
S/SR: Strain and strain rate
TAPSE: Tricuspid annular plane systolic excursion
TR PG: Tricuspid regurgitation peak gradient.

## Supplementary Materials

Supplementary Materials Table S1: Differences between the three functional groups by ANOVA. Table S2: Effect of high and low proBNP in Group A: participants with normal EF. Table S3: Effect of high and low proBNP in Group B: participants with reduced EF. Table S4: Effect of high and low proBNP in Group C: participants with atrial fibrillation. Table S5: Intra- and interobserver variability. Figure S1: Relation between NT-proBNP and the different atrial strain/SR measurements at corrected or noncorrected hypertension. The red line indicates the cut-off value for high NT-proBNP at 250 pg/ml. Figure S2: Relation between NT-proBNP and the different atrial strain/SR measurements in participants with the history of atrial fibrillation. The red line indicates the cut-off value for high NT-proBNP at 250 pg/ml.
