# Supplementary material for: Atrial Strain and Strain Rate in a General Population: Do These Measures Improve the Assessment of Elevated NT-proBNP Levels?
Source: Cardiol Res Pract. 2024 Aug 26;2024:1546629. doi: 10.1155/2024/1546629 (PMC11368553; doi:10.1155/2024/1546629)
Supplement: Supplementary Materials — Table S1: Differences between the three functional groups by ANOVA. Table S2: Effect of high and low proBNP in Group A: participants with normal EF. Table S3: Effect of high and low proBNP in Group B: participants with reduced EF. Table S4: Effect of high and low proBNP in Group C: participants with atrial fibrillation. Table S5: Intra- and interobserver variability. Figure S1: Relation between NT-proBNP and the different atrial strain/SR measurements at corrected or noncorrected hypertension. The red line indicates the cut-off value for high NT-proBNP at 250 pg/ml. Figure S2: Relation between NT-proBNP and the different atrial strain/SR measurements in participants with the history of atrial fibrillation. The red line indicates the cut-off value for high NT-proBNP at 250 pg/ml. [file 1546629.f1.zip › Supplementary material 2.docx]

| Table S1: Differences between the three functional groups by ANOVA | | | | |
| --- | --- | --- | --- | --- |
|  | EF ≥50% | EF <50% | Atrial Fibrillation |  |
|  | Group A | Group B | Group C |  |
| Group *n* | 1922 | 187 | 83 |  |
|  | Mean ± SD | Mean ± SD | Mean ± SD | p-value |
| PACS (%) | -20.5 ±6.0 | -18.0 ±7.0 * | -17.5 ±8.1* | <0.001 |
| ACS (%) | -26.1 ±12.6 | -19.5 ±10.6* | -21.9 ±13.1* | <0.001 |
| ATS (%) | -46.5 ±15.2 | -37.5 ±14.3* | -39.4 ±18.1* | <0.001 |
| Atrial SR S (1/s) | 2.4 ±1.0 | 2.1 ±1.0* | 2.0 ±1.0* | <0.001 |
| Atrial SR E (1/s) | -2.2 ±1.0 | -1.7 ±0.9* | -1.8 ±0.9* | <0.001 |
| Atrial SR A (1/s) | -2.9 ±1.0 | -2.6 ±1.1* | -2.5 ±1.2* | <0.001 |
| LASI (cm/s) | 0.24 ±0.16 | 0.34 ±0.27* | 0.50 ±1.10*† | <0.001 |
| MV E(cm/s) | 67 ±16 | 61 ±16* | 69 ±27*† | <0.001 |
| MV A (cm/s) | 64 ±15 | 68 ±16* | 65 ±18 | 0.003 |
| MV E/A (1/1) | 1.10 ±0.36 | 0.93 ±0.36* | 1.13 ±0.53† | <0.001 |
| MV DT (ms) | 191 ±48 | 196 ±47 | 273 ±49 | 0.140 |
| E/e´ | 9.7 ±3.2 | 11 ±4.2* | 11 ±6.4* | <0.001 |
| LA volume index (ml/m2) | 22.7 ±8z.4 | 21.5 ±8.2 | 26.3 ±14.3*† | <0.001 |
| TAPSE (cm) | 2.4 ±0.4 | 2.2 ±0.4 * | 2.3 ±0.5 | <0.001 |
| TR PG median (mmHg)‡ | 16.1 (5.5/20.2) | 13.6 (3.8/18.6)* | 17.4 (3.7/23.0)† | 0.004 |
| Velocity Basal Septal E (cm/s) | -6.5 ±2.1 | -5.1 ±1.8* | -5.8 ±2.1* | <0.001 |
| Velocity Basal Lateral E (cm/s) | -8.3 ±2.7 | -6.5 ±2.6* | -7.5 ±2.9*† | <0.001 |
| Velocity Basal Septal A (cm/s) | -7.5 ±1.7 | -7.2 ±1.8 | -6.6 ±2.6*† | <0.001 |
| Velocity Basal Lateral A (cm/s) | -6.9 ±2.5 | -6.4 ±2.9* | -6.4 ±2.9* | 0.002 |
| LV Stoke Volume (ml) | 56.8 ±5.4 | 43.2 ±5.5 | 53.6 ±6.8† | <0.001 |
| LV EF (%) | 128 ±32 | 140 ±41* | 143 ±41*† | <0.001 |
| LV EDV (ml) | 43 ±16 | 57 ±29* | 52 ±29* | <0.001 |
| LV ESV (ml) | 119 ±36 | 127 ±40* | 131 ±39* | 0.036 |
| LV mass Index (g/m^2^) | 86 ±22 | 83 ±22* | 91 ±23* | <0.001 |
| LV ES long Strain (%) | -20.2 ±2.7 | -17.5 ±3.4* | -19.3 ±4.4*† | <0.001 |
| LV PL SR S (1/s) | -1.19 ±0.20 | -1.11 ±0.22 * | -1.15 ±0.24 | <0.001 |
| LV PL SR E (1/s) | 1.56 ±0.34 | 1.29 ±0.33 * | 1.53 ±0.45† | <0.001 |
| LV PL SR A (1/s) | 1.11 ±0.27 | 1.14 ±0.31 | 1.07 ±0.30 | 0.192 |
| PACS: peak atrial contraction strain; ACS: atrial conduit strain; ATS: atrial total strain; SR: strain rate; S: during systole; E: during early filling phase; A: during atrial contraction; LASI: left atrial stiffness index (E/E´ /LA strain); MV: mitral valve; DT: deceleration time; LA: left atrium; TAPSE: tricuspid annular plane systolic excursion; TR PG: tricuspid regurgitation peak gradient; LV: left ventricle; EF: ejection fraction; EDV: end diastolic volume; ESV: end systolic volume; ES end systolic; PL: peak longitudinal  ANOVA with Bonferoni pairwise comparisons between groups with adjustment for multiple comparisons:  * p<0.05 for difference towards group A  † p<0.05 for difference towards group B  ‡Median (IQR) | | | | |

| Table S2 Effect of high and low proBNP in Group A: participants with normal EF | | | | | |
| --- | --- | --- | --- | --- | --- |
|  | Univariate Logistic Regression | | | Multiple Binary Logistic Regression | |
|  | Cases Positive/negative |  |  | 72/1688 |  |
| Group *n* |  | p-value | HR | p-value | HR (CI) |
| PACS (%) | 72/1710 | 0.430 |  |  |  |
| ACS (%) | 72/1710 | 0.007 | 1.032 (1.009-1058) | n.s. |  |
| ATS (%) | 72/1710 | 0.010 | 0.977 (0.959-0.994) | 0.031 | 0.978 (0.958-0.998) |
| Atrial SR S (1/s) | 72/1710 | 0.090 | 0.789 (0.599-1.038 | n.s. |  |
| Atrial SR E (1/s) | 72/1710 | 0.031 | 1.351 (1.027-1.776) | n.s. |  |
| Atrial SR A (1/s) | 72/1710 | 0.238 |  |  |  |
| LASI (1/%) | 72/1688 | 0.116 | 0.992 (0.982-1.002) | n.s. |  |
| MV E(cm/s) | 72/1693 | 0.219 |  |  |  |
| MV A (cm/s) | 72/1692 | 0.771 |  |  |  |
| MV E/A (1/1) | 72/1693 | 0.226 |  |  |  |
| MV DT (ms) | 72/1693 | <0.001 | 1.008 (1.004-1.012) | 0.002 | 1.007 (1.003-1.012) |
| E/e´ | 72/1693 | 0.028 | 0.001 (0.000-0.486) | 0.095 | 0.004 (0.000-2.477) |
| LA volume index (ml/m2) | 72/1716 | 0.363 |  |  |  |
| TAPSE (cm) | 72/1706 | 0.220 |  |  |  |
| TR PG median (mmHg) | 54/1464 | 0.224 |  |  |  |
| Velocity Basal Septal E (cm/s) | 72/1715 | 0.014 | 1.164 (1.031-1.314) | n.s. |  |
| Velocity Basal Lateral E (cm/s) | 72/1715 | 0.042 | 1.097 (1.004-1.200) | n.s. |  |
| Velocity Basal Septal A (cm/s) | 72/1715 | 0.512 |  |  |  |
| Velocity Basal Lateral A (cm/s) | 72/1715 | 0.073 | 0.917 (0.833-1.008) | n.s. |  |
| LV Stroke Vol (ml) | 72/1706 | 0.183 | 0.992 (0.981-1.004) | n.s. |  |
| LV EF (%) | 69/1543 | 0.432 |  |  |  |
| LV EDV (ml) | 72/1706 | 0.083 | 0.993 (0.985-1.001) | n.s. |  |
| LV ESV (ml) | 72/1706 | 0.101 | 0.986 (0.969-1.003) | n.s. |  |
| LV mass Index (g/m^2^) | 72/1704 | 0.434 |  |  |  |
| LV ES long Strain (%) | 65/1563 | 0.145 | 1.069 (0.977-1.170) | n.s. |  |
| LV PL SR S (1/s) | 65/1563 | 0.650 |  |  |  |
| LV PL SR E (1/s) | 65/1563 | 0.004 | 0.316 (0.145-0.688) | n.s. |  |
| LV PL SR A (1/s) | 65/1563 | 0.154 | 1.857 (0.793-4.345) | n.s. |  |
| PACS: peak atrial contraction strain; ACS: atrial conduit strain; ATS: atrial total strain; SR: strain rate; S: during systole; E: during early filling phase; A: during atrial contraction; LASI: left atrial stiffness index (E/E´ /LA strain); MV: mitral valve; DT: deceleration time; LA: left atrium; TAPSE: tricuspid annular plane systolic excursion; TR PG: tricuspid regurgitation peak gradient; LV: left ventricle; EF: ejection fraction; EDV: end diastolic volume; ESV: end systolic volume; ES end systolic; PL: peak longitudinal  Inclusion criteria for Stepwise Wald Logistic Regression was p<0.20  Significance in the final multiple binary logistic regression: p<0.05 | | | | | |

| Table S3 Effect of high and low proBNP in Group B: participants with reduced EF | | | | | |
| --- | --- | --- | --- | --- | --- |
|  | Univariate Logistic Regression | | | Multiple Binary Logistic Regression | |
|  | Cases Positive/negative |  |  | 9/110 |  |
| Group *n* |  | p-value | HR | p-value | HR (CI) |
| PACS (%) | 12/161 | 0.031 | 1.110 (1.010-1.221) | 0.007 | 1.44 (1.10-1.87) |
| ACS (%) | 12/161 | 0.179 | 1.046 (0.979-1.118) |  |  |
| ATS (%) | 12/161 | 0.038 | 0.947 (0.900-0.997) | n.s. |  |
| Atrial SR S (1/s) | 12/161 | 0.013 | 0.259 (0.089-0.755) | n.s. |  |
| Atrial SR E (1/s) | 12/161 | 0.064 | 2.466 (0.949-6.411) | n.s. |  |
| Atrial SR A (1/s) | 12/161 | 0.020 | 2.358 (1.145-4.857) | n.s. |  |
| LASI (1/%) | 11/156 | 0.666 |  |  |  |
| MV E(cm/s) | 11/157 | 0.171 | 0.045 (0.001-3.811) | n.s. |  |
| MV A (cm/s) | 11/157 | 0.546 |  |  |  |
| MV E/A (1/1) | 11/157 | 0.062 | 0.047 (0.002-1.169) | n.s. |  |
| MV DT (ms) | 11/59 | 0.285 |  |  |  |
| E/e´ | 11/157 | 0.190 | 0.001 (0.000-34.83) | n.s. |  |
| LA volume index (ml/m2) | 12/162 | 0.472 |  |  |  |
| TAPSE (cm) | 12/161 | 0.009 | 0.149 (0.035-0.627) | n.s. |  |
| TR PG median (mmHg)‡ | 10/136 | 0.139 | 0.938 (0.861-1.021) |  |  |
| Velocity Basal Septal E (cm/s) | 12/161 | 0.227 | 1.210 (0.957-1.530) | n.s. |  |
| Velocity Basal Lateral E (cm/s) | 12/161 | 0.111 |  |  |  |
| Velocity Basal Septal A (cm/s) | 12/161 | 0.352 |  |  |  |
| Velocity Basal Lateral A (cm/s) | 12/161 | 0.663 |  |  |  |
| LV Stoke Volume (ml) | 12/162 | 0.042 | 0.966 (0.935-0.999) | 0.010 | 0.91 (0.85-0.98) |
| LV EF (%) | 12/162 | 0.056 | 0.922 (0.849-1.002) | n.s. |  |
| LV EDV (ml) | 12/162 | 0.381 |  |  |  |
| LV ESV (ml) | 12/161 | 0.014 | 1.019 (1.004-1.035) | 0.011 | 1.06 (1.01-1.11) |
| LV mass Index (g/m^2^) | 12/154 | 0.461 |  |  |  |
| LV ES long Strain (%) | 12/140 | 0.015 | 1.186 (1.034-1.360) | n.s. |  |
| LV PL SR S (1/s) | 12/140 | 0.018 | 21.03 (1.704-259.4) | n.s. |  |
| LV PL SR E (1/s) | 12/140 | 0.064 | 0.255 (0.060-1.083) | n.s. |  |
| LV PL SR A (1/s) | 12/140 | 0.210 | 0.267 (0.034-2.111) | n.s. |  |
| PACS: peak atrial contraction strain; ACS: atrial conduit strain; ATS: atrial total strain; SR: strain rate; S: during systole; E: during early filling phase; A: during atrial contraction; LASI: left atrial stiffness index (E/E´ /LA strain); MV: mitral valve; DT: deceleration time; LA: left atrium; TAPSE: tricuspid annular plane systolic excursion; TR PG: tricuspid regurgitation peak gradient; LV: left ventricle; EF: ejection fraction; EDV: end diastolic volume; ESV: end systolic volume; ES end systolic; PL: peak longitudinal  Inclusion criteria for Stepwise Wald Logistic Regression was p<0.20  Significance in the final multiple binary logistic regression: p<0.05 | | | | | |

| Table S4: Effect of high and low proBNP in Group C: participants with atrial fibrillation | | | |
| --- | --- | --- | --- |
|  | Univariate Logistic Regression | | |
|  |  | p-value | HR (CI) |
|  | Case pos/neg |  |  |
| PACS (%) | 11/61 | 0.004 | 1.14 (1.04-1.25) |
| ACS (%) | 11/61 | 0.151 | 1.04 (0.98-1.11) |
| ATS (%) | 11/61 | 0.022 | 0.95 (0.91-0.98) |
| Atrial SR S (1/s) | 11/61 | 0.017 | 0.33 (0.14-0.82) |
| Atrial SR E (1/s) | 11/61 | 0.178 | 1.79 (0.77-4.15) |
| Atrial SR A (1/s) | 11/61 | 0.004 | 2.83 (1.40-5.71) |
| LASI (1/%) | 11/59 | 0.004 | 0.95 (0.92-0.95) |
| MV E(cm/s) | 11/59 | 0.123 | 5.24 (0.64-42.91) |
| MV A (cm/s) | 11/59 | 0.459 |  |
| MV E/A (1/1) | 8/59 | 0.013 | 4.30 (1.370-13.45) |
| MV DT (ms) | 11/59 | 0.633 |  |
| E/e´ | 11/59 | 0.392 |  |
| LA volume index (ml/m2) | 11/61 | 0.017 | 1.06 (1.01-1.11) |
| TAPSE (cm) | 11/60 | 0.030 | 0.21 (0.05-0.86) |
| TR PG median (mmHg)‡ | 9/57 | 0.092 | 1.06 (0.99-1.14) |
| Velocity Basal Septal E (cm/s) | 11/61 | 0.645 |  |
| Velocity Basal Lateral E (cm/s) | 11/61 | 0.809 |  |
| Velocity Basal Septal A (cm/s) | 11/61 | 0.043 | 1.26 (1.01-1.59) |
| Velocity Basal Lateral A (cm/s) | 11/61 | 0.055 | 1.25 (0.995-1.57) |
| LV Stoke Volume (ml) | 11/61 | 0.600 |  |
| LV EF (%) | 10/54 | 0.034 | 0.92 (0.84-0.99) |
| LV EDV (ml) | 10/61 | 0.359 |  |
| LV ESV (ml) | 10/61 | 0.153 | 1.01 (0.995-1.03) |
| LV mass Index (g/m^2^) | 11/60 | 0.664 |  |
| LV ES long Strain (%) | 7/52 | 0.389 | 1.07 (0.92-1.25) |
| LV PL SR S (1/s) | 7/52 | 0.717 |  |
| LV PL SR E (1/s) | 7/52 | 0.945 |  |
| LV PL SR A (1/s) | 7/52 | 0.062 | 0.07 (0.004-1.15) |
| PACS: peak atrial contraction strain; ACS: atrial conduit strain; ATS: atrial total strain; SR: strain rate; S: during systole; E: during early filling phase; A: during atrial contraction; LASI: left atrial stiffness index (E/E´ /LA strain); MV: mitral valve; DT: deceleration time; LA: left atrium; TAPSE: tricuspid annular plane systolic excursion; TR PG: tricuspid regurgitation peak gradient; LV: left ventricle; EF: ejection fraction; EDV: end diastolic volume; ESV: end systolic volume; ES end systolic; PL: peak longitudinal  Patients had atrial fibrillation defined by Hx or ECG | | | |

| Table S5: Intra- and interobserver variability | | | | |
| --- | --- | --- | --- | --- |
| N=884 | | Intra-Class-Correlation Coefficient | Lower limit | Upper limit |
| Intraobserver | PACS (%) | 0.845 | 0.701 | 0.917 |
|  | ACS (%) | 0.872 | 0.617 | 0.945 |
|  | ATS (%) | 0.741 | 0.526 | 0.859 |
|  | Atrial SR S (1/s) | 0.709 | 0.398 | 0.852 |
|  | Atrial SR E (1/s) | 0.863 | 0.570 | 0.942 |
|  | Atrial SR A (1/s) | 0.779 | 0.578 | 0.892 |
| Interobserver | PACS (%) | 0.748 | 0.433 | 0.877 |
|  | ACS (%) | 0.822 | 0.556 | 0.917 |
|  | ATS (%) | 0.822 | 0.556 | 0.917 |
|  | Atrial SR S (1/s) | 0.783 | 0.493 | 0.896 |
|  | Atrial SR E (1/s) | 0.787 | 0.488 | 0.899 |
|  | Atrial SR A (1/s) | 0.807 | 0.429 | 0.917 |
|  | | | | |

Figure S1: Relation between NT-proBNP and the different atrial strain/SR measurements at corrected or non-corrected hypertension. The red line indicates the cut-off value for high NT-proBNP at 250 pg/ml.

Figure S2: Relation between NT-proBNP and the different atrial strain/SR measurements in participants with the history of atrial fibrillation. The red line indicates the cut-off value for high NT-proBNP at 250 pg/ml.
